# Feasibility of Apatinib in Radioiodine-Refractory Differentiated Thyroid Carcinoma

**DOI:** 10.3389/fendo.2022.768028

**Published:** 2022-02-23

**Authors:** Wei Du, Xiangyu Shi, Qigen Fang, Xu Zhang, Shanting Liu

**Affiliations:** ^1^Department of Head Neck and Thyroid, Affiliated Cancer Hospital of Zhengzhou University, Henan Cancer Hospital, Zhengzhou, China; ^2^Department of Breast and Thyroid Surgery, People’s Hospital of Changshou District, Chongqing, China

**Keywords:** differentiated thyroid carcinoma, apatinib, radioiodine refractory differentiated thyroid carcinoma, targeted treatment, drug efficacy

## Abstract

**Objectives:**

Our aim was to describe our experience in using apatinib as treatment for radioiodine-refractory differentiated thyroid carcinoma (RAIR-DTC).

**Methods:**

Forty-seven patients undergoing apatinib treatment for RAIR-DTC were prospectively enrolled in this study. The study endpoints were objective response rate (ORR), disease control rate (DCR), progression-free survival (PFS), overall survival (OS), and rate of adverse events.

**Results:**

No patients achieved complete response, while 36 (76.6%) and 8 (17.0%) patients achieved partial response and stable disease, respectively. The ORR and DCR were 76.6% and 93.6%, respectively. The median PFS and OS were 18 and 59 months, respectively. A total of 91 adverse events occurred, of which 21 were graded as grade 3 or higher. There were no drug-related deaths.

**Conclusions:**

Apatinib has distinct anti-RAIR-DTC efficacy in terms of ORR, PFS, and OS and has a favorable safety profile. It is a feasible treatment option for RAIR-DTC.

## Introduction

Differentiated thyroid carcinoma (DTC) is the most common endocrine malignancy. The mainstay of treatment is surgical resection, occasionally followed by iodine-131 therapy (1). Most patients treated accordingly have good prognoses. However, disease recurrence may occur in a minor subset of DTC patients (2). If complete surgical excision is not possible, or if tumor progression has occurred and distant metastases are present, iodine-131 treatment is used (3). Nevertheless, some recurrent/metastatic lesions lose their ability of iodine concentration, which is known as radioiodine-refractory DTC (RAIR-DTC) (4). RAIR-DTC is associated with poor survival and contributes to most DTC-related deaths (5).

Treatment of RAIR-DTC is challenging in clinics as the therapeutic options are limited (6). Sorafenib and lenvatinib were approved for the treatment of progressive, metastatic RAIR-DTC by the European Medicines Agency (EMA) and the United States Food and Drug Administration (FDA), owing to the clinical benefits of these drugs reported in two phase III clinical trials (7, 8). However, both drugs are too expensive for most Chinese patients. Apatinib (Jiangsu Hengrui Medicine, Lianyungang, China) is another small-molecule tyrosine kinase inhibitor that has similar targets to sorafenib and lenvatinib. The effectiveness of apatinib has been demonstrated in a phase II trial by Lin et al., who reported an objective response rate (ORR) of 80% and a disease control rate (DCR) of 95% (9). Zhang et al. (10) have recently reported a case of inoperable DTC treated with apatinib. Research works regarding the application of apatinib in DTC are limited. Therefore, in the current study, we aimed to describe our experience in using apatinib for the treatment of RAIR-DTC.

## Patients and Methods

### Ethical Considerations

This study was approved by the Henan Cancer Hospital Institutional Research Committee. All participants provided written informed consent. All procedures involving human participants were conducted according to the principles of the Declaration of Helsinki.

### Patient Enrollment

**Figure 1:**
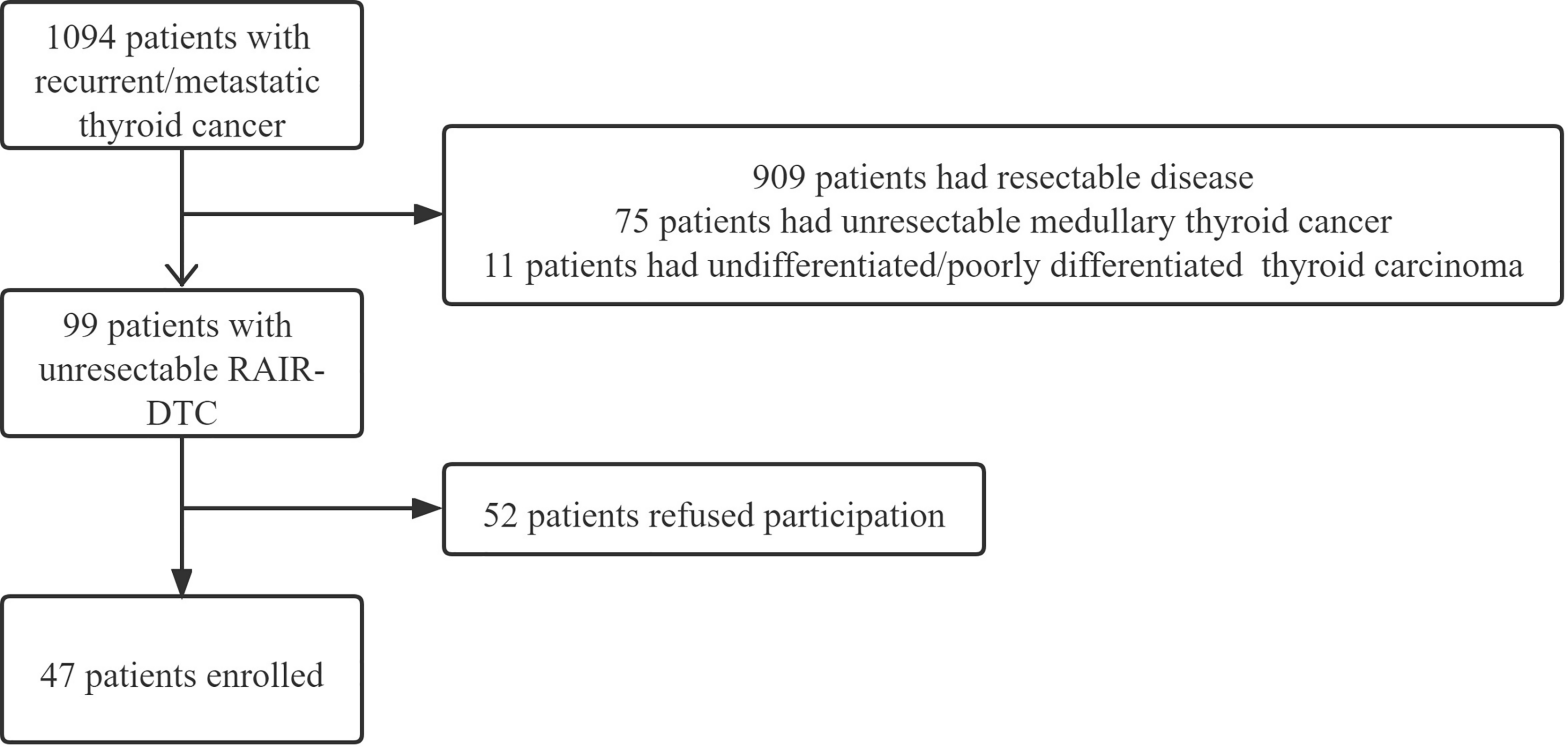
Flowchart of the enrollment of radioiodine-refractory differentiated thyroid carcinoma (RAIR-DTC) patients.

From January 2015 to December 2020, a total of 1,094 patients with recurrent/metastatic thyroid cancer presented to our department in need of medical treatment. Among them, 99 patients were confirmed as having unresectable RAIR-DTC based on the following criteria: no uptake of iodine in the target lesion; progressive disease 12–16 months after iodine-131 therapy; and progressive disease after receiving a cumulative iodine-131 dose of above 22.3 GBq (11). All of the 99 patients had at least one measurable lesion and had not received prior tyrosine kinase inhibitor treatment or chemotherapy. After the potential benefits and complications were clearly explained, 30 patients refused enrollment owing to the possibility of adverse complications, 12 patients refused enrollment due to poor economy, and 10 patients abandoned further treatment of their metastatic thyroid cancer prior to initiating apatinib. A total of 47 patients finally agreed to participate in our study (**Figure 1**).

### Study Design

The 47 participants received treatment of 500 mg of apatinib daily for a 4-week cycle. The patients were followed every 4 weeks for the first 8 weeks and every 8 weeks thereafter with CT or MRI imaging. The dosage of apatinib was reduced in the instance of a grade 3 or higher adverse event. Treatment continued until the occurrence of disease progression, drug intolerance, or withdrawal of consent to participate in the study.

### Endpoints

The primary endpoints in this study were ORR and DCR. The ORR refers to the percentage of patients who showed complete response (CR) or partial response (PR), while the DCR refers to the percentage of patients who showed CR, PR, or stable disease (SD), according to the Response Evaluation Criteria in Solid Tumours (RECIST) 1.1 (12). The secondary endpoints included progression-free survival (PFS) and overall survival (OS). PFS was calculated from the date of initial treatment to the date of disease progression or death from any cause. OS was calculated from the date of initial treatment to the date of the last follow-up or death from any cause (13). The third endpoint was the rate of adverse reactions, which were graded according to the National Cancer Institute Common Terminology Criteria for Adverse Events (version 4.0) (14).

### Statistical Analyses

All statistical analyses were performed using SPSS version 20.0 (IBM Inc., Chicago, IL, USA), and the Kaplan–Meier method was used to calculate the survival rates. Statistical significance was defined as *p* < 0.05.

## Results

### Data

Of the 47 participants enrolled in our study, 19 (40.4%) were men and 28 (59.6%) were women, and the mean age was 55.8 years (range = 48–68 years). Diagnosis of papillary thyroid carcinoma was confirmed with fine needle aspiration, and the mean time between previous treatment and diagnosis of RAIR-DTC was 2.5 ± 1.2 years. Thirty-two (68.1%) patients were found to have the *BRAF^V600E^* mutation. The Eastern Cooperative Oncology Group (ECOG) performance status scores were 0 in 15 (31.9%) patients, 1 in 30 (63.8%) patients, and 2 in 2 (4.3%) patients. All patients had previously received surgical treatment and iodine-131 therapy, and the mean cumulative dose of iodine-131 was 406 ± 116 mCi. Distant metastases were present in all patients, namely, lung metastasis in 35 (74.5%), bone metastasis in 16 (34.0%), liver metastasis in 7 (14.9%), and brain metastasis in 4 (8.5%) cases (**Table 1**).

**Table 1:** Demographic information of the 47 patients.

Variable	Number (%)
Age (years)	
≤50	3 (6.4)
>50	44 (93.6)
Sex	
Male	19 (40.4)
Female	28 (59.6)
Papillary thyroid carcinoma	47 (100)
*BRAF^V600E^* mutation	32 (68.1)
ECOG	
0	15 (31.9)
1	30 (63.8)
2	2 (4.3)
Cumulative dose of iodine-131	406 ± 116 mCi
Distant metastasis	
Lung	35 (74.5)
Bone	16 (34.0)
Liver	7 (14.9)
Brain	4 (8.5)

ECOG, Eastern Cooperative Oncology Group.

### ORR and DCR

**Figure 2:**
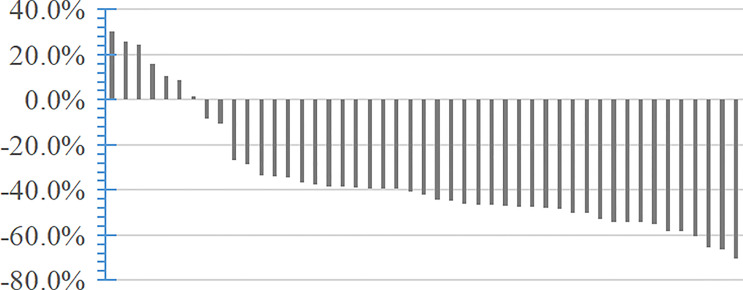
Treatment responses of the 47 study participants.

During apatinib treatment, no patients achieved CR, while 36 (76.6%) and 8 (17.0%) patients achieved PR and SD, respectively; progressive disease occurred in 3 (6.4%) patients (**Figure 2**, **Supplementary Data**, and **Supplementary Figure S1**). The ORR and DCR were 76.6% and 93.6%, respectively. The mean time to objective response was 2.5 ± 1.4 months, and the mean duration of response was 17.7 ± 8.6 months.

Dose reduction was necessary for 20 patients. Of these 20 patients, PR, SD, and progressive disease occurred in 14, 5, and 1, respectively. Two patients withdrew from this study because of serious adverse reaction and disease progression.

### PFS and OS

**Figure 3:**
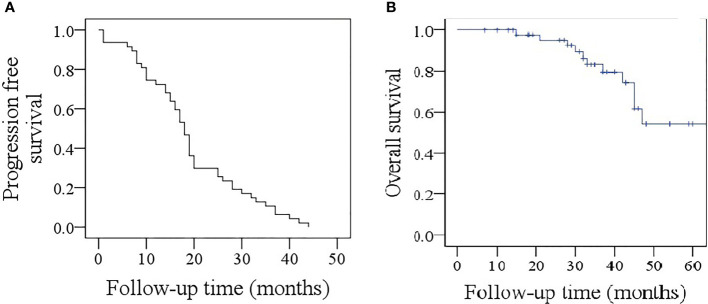
Progression-free survival **(A)** and overall survival **(B)** of the 47 study participants.

At the last follow-up, with a median time of 35 months, all patients showed disease progression, and the median PFS was 18 months (**Figure 3A**). In patients with the *BRAF^V600E^* mutation, the median PFS was 18 months. This was comparable to the PFS of 16 months in patients without the *BRAF^V600E^* mutation (*p* = 0.567).

A total of 11 patients died, and the median OS was 59 months (**Figure 3B**). In patients with the *BRAF^V600E^* mutation, the median OS was 59 months, which was the same as that of patients without the *BRAF^V600E^* mutation (*p* = 0.554).

### Adverse Reactions

Ninety-one adverse events occurred in the 47 patients, of which 21 events were of grade 3 or higher. There were no drug-related deaths. The most common hematological toxicity was leucopenia, which occurred in 10.6% of patients. The three most common non-hematological toxicities were hypertension, proteinuria, and hand–foot skin reaction, which accounted for nearly 50% of all adverse events and 70% of the grade 3 and 4 adverse events (**Table 2**).

**Table 2:** Adverse events in the 47 patients undergoing apatinib for radioiodine-refractory thyroid cancer.

Adverse events	Number	
	All grades	Grades 3/4
Hematologic toxicity		
Leucopenia	5	1
Granulocytopenia	3	1
Thrombocytopenia	2	1
Non-hematologic toxicity		
Hypertension	20	5
Proteinuria	19	5
Hand–foot skin reaction	16	4
Diarrhea	6	2
Liver dysfunction	5	2
Fatigue	5	0
Emesia	4	0
Nausea	3	0
Bleeding	2	0
Bellyache	1	0

## Discussion

In this study, we aimed to determine the efficacy of apatinib in RAIR-DTC. We found that apatinib had reliable oncologic efficacy in RAIR-DTC patients, with limited adverse events.

RAIR-DTC is relatively uncommon, as shown in a previous study in which it only occurred in 2.2% of the 5,163 DTC patients treated (4). Nonetheless, RAIR-DTC was associated with a low 10-year OS rate (10%) (15); therefore, there is a critical need for effective treatment. Sorafenib was the first tyrosine kinase inhibitor approved by the FDA for RAIR-DTC. In the phase III DECISION trial, the sorafenib arm had an ORR of 12% and a longer PFS (by 5 months) compared with the placebo arm (7). Lenvatinib was the second tyrosine kinase inhibitor approved by the FDA and EMA for RAIR-DTC. In the phase III SELECT study, the lenvatinib arm showed an ORR of 64.8% and a median PFS of 18.3 months, both of which were higher than those of patients receiving placebo (8). A recently published phase III study has revealed the high efficacy of lenvatinib in controlling RAIR-DTC in Chinese patients (16). However, sorafenib has only been approved for advanced renal cell carcinoma and inoperable hepatocellular carcinoma in China. Since both sorafenib and lenvatinib are expensive, other feasible strategies are needed for RAIR-DTC therapy.

Apatinib, another tyrosine kinase inhibitor, is made in China. It was first used for the treatment of advanced gastric or gastroesophageal junction adenocarcinoma. Patients treated with apatinib showed significant improvements in PFS and OS (17). The application of apatinib to RAIR-DTC has only been explored by Lin et al. (9, 10, 18–20). Lin et al. reported overall ORR and DCR of approximately 80% and 90%, respectively. This finding is consistent with the findings of the present study. The ORR and DCR in our study were significantly higher than those in the DECISION trial and slightly higher than those in the SELECT study. There are at least three considerations that may explain the differences in the findings: 1) research has shown that the migration and proliferation of endothelial cells are significantly regulated by vascular endothelial growth factor receptor-2 (VEGFR-2) (21); 2), apatinib has shown highly selective VEGFR-2 inhibition, with an IC_50_ of 1 nM *in vitro*, which is apparently lower than the IC_50_ values of lenvatinib and sorafenib (18, 22); and 3) the demographic and genomic differences between the Western and Chinese populations might also be partially responsible for the differences in the findings between studies.

The median PFS and OS in this study were 18 and 59 months, respectively. These findings are consistent with those in the study by Lin et al. (9). Koehler et al. (23) recently conducted a study in the real-world setting, at six German referral centers, and found median PFS rates of 9 and 12 months with the use of sorafenib and lenvatinib, respectively; the median OS rates with the use of sorafenib and lenvatinib were 37 and 47 months, respectively. Ahn et al. (24) showed that the median OS and PFS of 40 patients treated with sorafenib were 34.3 and 14.8 months, respectively. Apatinib appears to have the most oncological benefits among the three tyrosine kinase inhibitors; however, direct comparisons of apatinib, sorafenib, and lenvatinib are impossible due to the differences in patient characteristics and follow-up frequencies, among other factors.

The time to objective response is an important factor to consider when analyzing drug efficacy. We found that the time to objective response with apatinib treatment was 2.5 ± 1.4 months, which was shorter than that in the study by Lin et al. (9). A possible explanation is the difference in the inclusion standards between the studies: poorly differentiated thyroid cancer patients were also enrolled in the study by Lin et al. More importantly, the time to objective response found in our study was shorter than the time of 20% additional tumor volume increase in the placebo arms of the DECISION and SELECT trials (8, 9). This shows the potential role of apatinib in neoadjuvant treatment due to its rapid tumor burden reduction effect. Zhang et al. (10) found that an inoperable tumor shrank from 56 × 37 to 29 × 26 mm after 6 weeks of apatinib treatment; the tumor could then be completely excised with minimal complications.

The pathogenic role of the *BRAF^V600E^* mutation in DTC is well established (25). Lin et al. (9) noted that patients with the *BRAF^V600E^* mutation had longer PFS than those without the *BRAF^V600E^* mutation. A similar finding was also described by Brose et al. (7). However, in our study, we did not detect this association, most likely due to our limited sample size.

Drug safety is a key issue that requires attention. In this study, although all patients experienced adverse events, most of them were of grade 1 or 2, and there were no drug-related deaths. Lin et al. (18) reported that the toxicity of apatinib was associated with the dose: the 500-mg dose protocol achieved similar oncological control and was associated with fewer adverse events compared with the 750-mg dose protocol; this concept was also applied in the present study. In the DECISION and SELECT trials, the rates of grade 3 and 4 adverse events for the use of sorafenib and lenvatinib were 82.1% and 75.9%, respectively (5). In a recent trial involving Chinese patients, the rate of grade 3 and 4 adverse events was 85.4%, with 10 fatal treatment-related adverse events (16). This finding suggests the high safety profile of apatinib for RAIR-DTC treatment.

In conclusion, apatinib has distinct anti-RAIR-DTC efficacy in terms of ORR, PFS, and OS and has a favorable safety profile. It is a feasible treatment option for RAIR-DTC.

## Data Availability Statement

The datasets presented in this study can be found in online repositories. The names of the repository/repositories and accession number(s) can be found in the article/**Supplementary Material**.

## Ethics Statement

This study was approved by Henan Cancer Hospital Institutional Research Committee. All participants signed a written informed consent agreement. The patients/participants provided their written informed consent to participate in this study.

## Author Contributions

All authors made a contribution to the study design, manuscript writing, study selection, data analysis, study quality evaluation, and manuscript revision. All authors have read and approved the final manuscript.

## Conflict of Interest

The authors declare that the research was conducted in the absence of any commercial or financial relationships that could be construed as a potential conflict of interest.

## Publisher’s Note

All claims expressed in this article are solely those of the authors and do not necessarily represent those of their affiliated organizations, or those of the publisher, the editors and the reviewers. Any product that may be evaluated in this article, or claim that may be made by its manufacturer, is not guaranteed or endorsed by the publisher.
