# Supplementary material for: Feasibility of Apatinib in Radioiodine-Refractory Differentiated Thyroid Carcinoma
Source: Front Endocrinol (Lausanne). 2022 Feb 23;13:768028. doi: 10.3389/fendo.2022.768028 (PMC8904562; doi:10.3389/fendo.2022.768028)
Supplement: Supplementary file 1 [file DataSheet_1.doc]

Supplemental data

The mean number of cycles of apatinib taken by the RAIR-DTC patients was 16.7±6.5, and the number of cycles at which partial response occurred was 2.4 ± 1.5.

Supplemental Figure 1 legend: A: the metastatic tumors in the mediastinum before treatment; B: the metastatic tumors in the mediastinum after treatment.
