# Supplementary figures and images for: Feasibility of Apatinib in Radioiodine-Refractory Differentiated Thyroid Carcinoma
Source: Front Endocrinol (Lausanne). 2022 Feb 23;13:768028. doi: 10.3389/fendo.2022.768028 (PMC8904562; doi:10.3389/fendo.2022.768028)

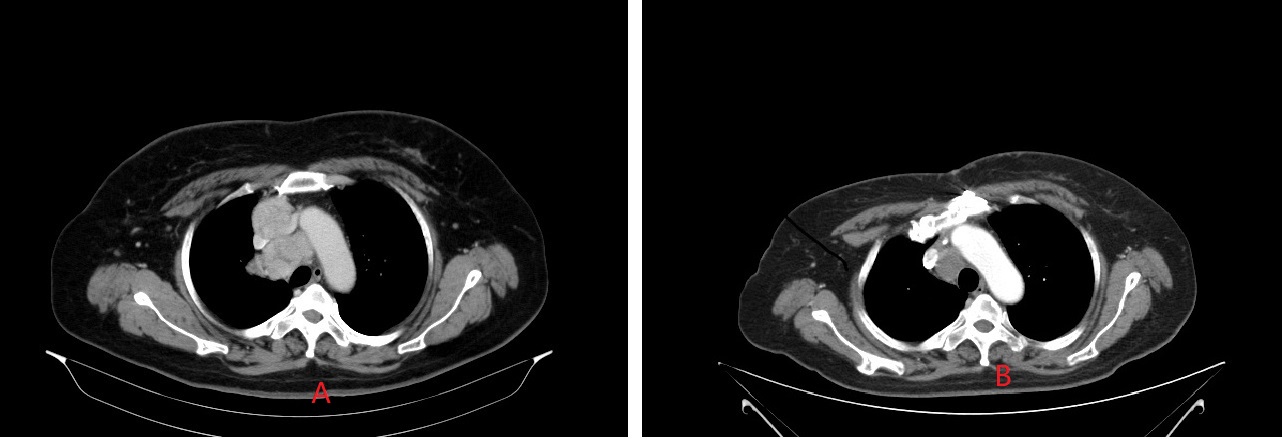

Supplement: Supplementary file 2 [file Image_1.jpeg]
